# Altered Morphologies and Functions of the Olfactory Bulb and Hippocampus Induced by miR-30c

**DOI:** 10.3389/fnins.2016.00207

**Published:** 2016-05-09

**Authors:** Tingting Sun, Tianpeng Li, Henry Davies, Weiyun Li, Jing Yang, Shanshan Li, Shucai Ling

**Affiliations:** ^1^Institute of Neuroscience and Anatomy, Zhejiang University School of MedicineHangzhou, China; ^2^College of Environment Science and Engineering, Donghua UniversityShanghai, China; ^3^Institute of Neuroscience, Zhejiang University School of MedicineHangzhou, China

**Keywords:** adult neurogenesis, miR-30c, semaphorin3A, olfaction, memory, olfactory bulb, dentate gyrus

## Abstract

Adult neurogenesis is considered to contribute to a certain degree of plasticity for the brain. However, the effects of adult-born neurons on the brain are still largely unknown. Here, we specifically altered the expression of miR-30c in the subventricular zone (SVZ) and dentate gyrus (DG) by stereotaxic injection with their respective up- and down-regulated lentiviruses. Results showed an increased level of miR-30c enhanced adult neurogenesis by prompting cell-cycles of stem cells, whereas down-regulated miR-30c led to the opposite results. When these effects of miR-30c lasted for 3 months, we detected significant morphological changes in the olfactory bulb (OB) and lineage alteration in the hippocampus. Tests of olfactory sensitivity and associative and spatial memory showed that a certain amount of adult-born neurons are essential for the normal functions of the OB and hippocampus, but there also exist redundant newborn neurons that do not further improve the functioning of these areas. Our study revealed the interactions between miRNA, adult neurogenesis, brain morphology and function, and this provides a novel insight into understanding the role of newborn neurons in the adult brain.

## Introduction

Adult neurogenesis of rodent is mainly derived from two regions: the SVZ bordering the lateral ventricles and the DG of the hippocampus, which produce a stable supply of newborn neurons to maintain normal function of the brain. These newborn neurons later mature and integrate in their target regions and function respectively as inhibitory and excitatory interneurons in the OB and hippocampus (Kriegstein and Alvarez-Buylla, 2009; Kelsch et al., 2010; Kempermann et al., 2015). Despite extensive cellular and electrophysiological characterization of individual adult newborn neurons, the effects of adult neurogenesis on the morphologies and functions of the brain are largely unknown (De Marchis and Puche, 2012). There still exists controversy with regards to the effects of adult neurogenesis on the functions of the OB and hippocampus (Imayoshi et al., 2008; Yau et al., 2015), though emerging reports are revealing the interactions of these newborn neurons and their connected neurons in their local circuits (Drew et al., 2013; LaSarge et al., 2015).

miRNAs, the small length of the noncoding RNAs, are critical post-transcriptional regulators that inhibit the transcription or induce degradation of their target mRNAs (Davis et al., 2015). Previous study reports that some miRNAs play significant roles in modulating the proliferation and differentiation of neural stem cells (Wakabayashi et al., 2014). However, the roles of miRNAs in adult neurogenesis are yet to be investigated. miR-30c is an abundant miRNA in the brain. It has been confirmed to be the regulator of self-renewal and differentiation of glioma cells *in vitro* (Chao et al., 2015).

To investigate the effects of miR-30c on the morphologies and functions of the OB and hippocampus, we specifically up- or down-regulated the level of miR-30c in the SVZ and DG by stereotaxic injection and then detected changes in brain morphology and associative and spatial memory.

## Materials and methods

### Animals

Male C57BL/6 mice at 8–20 weeks of age were used for this study. Mice were obtained from Shanghai Laboratory Animal Center (SLAC, Chinese Academy of Science, Shanghai, China). Animal care and use were performed in accordance with the guidelines of the China Committee on Animal Experiments and with the approval of Zhejiang University Animal Care and Use committee. All mice were housed in a central facility and maintained under controlled conditions of normal humidity and temperature, with standard alternating 12 h periods of light and darkness. Animals had free access to water and food. All behavioral experiments were performed by the same experimenters and *in vivo* procedures were performed with mice under deep anesthesia with 3% pentobarbital sodium (70 mg/kg, P3761, Sigma-Aldrich).

### Vector construction, lentivirus production, and titration

Construction of miR-30c-overexpression (miR-30c-OE) vector was performed as previously described (Sun et al., 2014). In brief, fragments of miR-30c precursors were obtained by PCR amplification of genomic DNA of C57BL/6 mice with specific primers (Table 1). Then the double sequences were subcloned into the lentiviral vector of pLVX-tdTomato (Clontech). Sequences of miR-30c-knockdown (miR-30c-KD) were synthesized (Generay Biotech Co., Shanghai, China; Table 1) and then these sequences were subcloned into the *EcoR*I and *BamH*I site of the pLVX-EGFP (Clontech; Gentner et al., 2009). A blank lentiviral vector of pLVX-EGFP was used as the control. Lentivirus production was carried out by cotransfected each above vector with pSPAX2 and PMD2.G in the packaging cell line 293T (Invitrogen) and harvested lentivuses from the supernatant 48 h after transfection. The lentirivus stocks were diluted in six gradient and then these gradients were added into six-well plate with 1–4 × 10^5^ 293T cells/well. Fluorescent cells were calculated by flow cytometry. Transducing Units (TU) were calculated by the algorithm: TU/ml = seeding cell number × percentage of fluorescent cell number × 1000 × 1/added volume of lentivirus stock (μl).

**Table 1:** **Primers of miR-30c precursor and sequences of miR-30c-OE and miR-30c-KD**.

**ID**	**Forward primer(5′–3′)**	**Reverse primer(5′–3′)**	
mmu-mir30c-2_OE	*CGCGGATCC*ATTGATTAGGCATCAAG	*CCGGAATTC*TGGGATTATAGGCACACA
ID	Sequence(5′–3′)	
miR-30-KD	GCTGAGAGTGTGAAGTGTTTACAGCATCGGCTGAGAGTGTGAAGTGTTTACACGATCGGCTGAGAGTGTGAAGTGTTTACAGCATCGGCTGAG AGTGTGAAGTGTTTACACGATCGGCTGAGAGTGTGAAGTGTTTACAGCATCGGCTGAGAGTGTGAAGTGTTTACACGATCGGCTGAGAGTGTGAA GTGTTTACAGCATCGGCTGAGAGTGTGAAGTGTTTACA	
miE-30c-OE	ATTGATTAGGCATCAAGAAGGTAAGCATCCTGCAGCCTTCTTACCGGCTCCAGACCTAGAAGCTGAAATTTAGTCCCAAGTACACTCTGTT TTCTAGCCCCTGTTCTAACACGTCACGGGATCTCCAGATGTTCCACGGCATCCTGATGCTTGTGCTGCTCACCGCCAACCTGCTCTA GAGAGCACTGAGTGACAGATATTGTAAACATCCTACACTCTCAGCTGTGAAAAGTAAGAAAGCTGGGAGAAGGCTGTTTACTCTCTCTGCCTT GGAAATCAGCTAAGAGAAATGAATTTTAGATGGCCTATAGGTGATCCTATCAAAGATCTGTTATGCGGTCAACTCTAAAAGGTAAAAGGGTGAGCT AATCTGTGCTAAGAAGTACAAGACACTTTGGGAGATTGGTTATTTTTAACTGTTTAAGAAGACCAAGTATGGTGGTGTGTGCCTATAATCCCA	

*Italic, endogenous enzymes cleavage sites; underline in miR-30c-OE indicates the precursor sequence of miR-30c; underlined nucleotides in miR-30c-KD, nucleotide linkers; the other nucleotides in miR-30c-KD, sequences that complementary binding with the mature sequences of miR-30c*.

### Brain stereotaxic injection

Eight-week-old male C57BL6/J mice were used for stereotaxic injections. All surgeries were performed using aseptic technique. Mice were anesthetized with pentobarbital sodium (70 mg/kg, P3761, Sigma-Aldrich), placed into a stereotaxic frame (RWD Life Science Co. Ltd, Shenzhen, China), and unilaterally injected with ~1 × 10^6^ TU lentiviruses (miR-30c-OE, miR-30c-KD or pLVX-EGFP), either in the SVZ (bregma 0.86 mm; lateral 0.8 mm; ventral 3.8 mm), or the DG (bregma−1.75 mm; lateral 0.75 mm; ventral 2.3 mm; Paxinos and Franklin, 2001). Mice were allowed to recover for 4 weeks before performing behavioral experiments. Placements of stereotaxic injections were verified 2 weeks after the experiments by detecting fluorescence of the tagged fluorescence proteins. Fluorescent images were acquired using a confocal microscope (FV-1000, Olympus, Japan).

### Food pellet buried experiment

A scent food pellet (grape cookie, diameter ~ 0.5 cm) was placed at random in cage (25 × 25 × 25 cm^3^) and the latency to find the food pellet was defined as the time between when the mouse was placed in the cage and when the mouse found the food pellet and grasped or bit it. Food was restricted for mice (0.2 g of food per mouse/24 h) 1 day before the test to evoke mice's desire to find the food pellet. To better track the trajectory of mice, infrared video was installed below the testing cage. The food pellet was buried ~0.5 cm below the surface of a 3 cm deep layer of mouse bedding material. One test per day was performed and the threshold for the animal to find the food pellet was 5 min. An animal that did not find the food pellet within 5 min was removed and placed back into its home cage. The bedding in the test chamber was changed between trials.

### Conditional fear memory examination

Conditional fear memory examination was performed based on pavlovian effects that the aversive stimulus was memorized with a neutral stimulus by associative memory. It is a test widely used to detect the hippocampus-dependent associative learning and memory (Lin et al., 2015). The conditioning was performed in two behavior chambers (Med Association Inc., Vermont, USA). Each chamber consisted of an isolation cabinet equipped with a computer-controlled light and sound exposure system, and there was an open door between them through which the mice can move freely between the two chambers. The bottom of the enclosure contained steel bars capable of delivering electric shocks. The conditioning system is equipped with an infrared scanning light that can recognize the location of the mice, and only one of chambers that the mouse stands on can emit the light, sound and shock stimuli. The conditioned stimulus (auditory presentations, 72 dB, white noise, 5 s) and light are co-started and co-terminated during the trial. Then an electric shock followed (unconditioned stimulus, US, 0.4 mA foot shock, 15 s). If a mouse escaped to the other chamber during the 5 s condition stimulus stage, this trial was defined as “escape,” otherwise it was marked as “latency.” Each day 30 trials were held for each mouse and the test lasted for 4 days with an interval of 24 h. The long-term memory test was performed on the 9th day.

### Morris water maze test

Morris water maze (MWM, Med Association Inc., Vermont, USA) is a standard way to study the learning and memory of animals. The MWM consists of a circular water tank (120 cm in diameter, 50 cm in height) that is divided into four quadrants. The recording device includes one camera suspended 3 m directly above the pool and a computer system with ANY-maze Video Tracking System software. A platform (10 cm in diameter and 28 cm in height) is hidden 1 cm below the water surface in the center of one of the quadrants. Animals were trained in four trials per day. For each trial, mice were placed into the water in one of the four quadrants, facing the wall. The time required for the animal to find the hidden platform was recorded as escape latency. A trial was terminated once the mouse found the platform. If the mouse failed to find the platform within 90 s, it was guided to the platform and allowed to stay for 20 s, and a value of 90 s was assigned as the escape latency. The test lasted for 6 days and on the 6th day, each mouse was put into water at a fixed point after the platform had been removed. The frequency of the mouse crossing the place where the platform was previously located within 90 s was then recorded.

### 5-bromo-2′-deoxyuridine (BrdU) labeling and frozen slice preparation

Three weeks after stereotaxic injection, mice were given intraperitoneal injections of BrdU (50 μg/g body weight, Sigma) and 4 h later they were sacrificed for detecting the newborn cells in the SVZ. To evaluate the DG newborn cells, mice were given one daily injection of BrdU for 7 days (*n* = 4 mice/group). Sectioning of the brain was carried out as in a previous report with minor modifications (Encinas and Enikolopov, 2008). Six sets of sagittal sections (for analyzing the hippocampus) from each mouse were obtained by cutting the right hemisphere in the lateral to medial direction. The same number sets of coronal sections (for analyzing the SVZ) were obtained by cutting the right hemisphere from anterior to posterior. Each set provided a representative sample of the hippocampus and the SVZ. The first set was selected for calculation (15 slices) and the slice width was 20 μm.

### The whole OB morphological analysis

Animals (3 mice/group) were deeply anesthetized with sodium pentobarbital. The brains containing the OB were carefully separated from the cranium and then rinsed in 0.1 M PBS for 1 min and placed in line for photography.

### Immunofluorescence and analysis

The primary antibodies used were mouse anti-BrdU (B8434, Sigma-Aldrich), rabbit anti-glial fibrillary acidic protein (GFAP; ARH4195, AR), mouse anti-Nestin (MAB353, Millipore), rabbit anti-Neuropilin-1 (NP1; ab81321, Abcam). The experiment was conducted according to standard procedures. Slices were first denatured in 2 N HCl at 37°C for 1 h and neutralized by 0.1 M borate for newborn cell detection in SVZ and DG slices were treatment with 10 mM sodium citrate for antigen-retrieval (Tang et al., 2007). Then, brain sections were incubated with a blocking and permeabilization solution (PBS containing 1% Triton-100X and 3% goat serum) for 1 h at room temperature and incubated overnight at 4°C with the primary antibodies. The second antibodies were AlexaFluor 488 donkey anti-rabbit IgG (CA21206s, Invitrogen), AlexaFluor 594 donkey anti-mouse IgG (CA21203s, Invitrogen) and they were incubated for 2 h at room temperature. DAPI (4'6-diamidino-2-phenylindole, D21490, Molecular Probes) was used to stain nuclei. After washing with PBS, the sections were mounted with fluorescent mounting medium (Dako Cytomation) and detected under a fluorescence microscope or confocal microscope (FV-1000, Olympus, Japan).

The first set of sagittal sections and coronal sections were selected for counting the BrdU-positive cells in the hippocampus and the SVZ, respectively. The second sets of sections were used to calculate the width of granular cell layers and glomerular layers. Images were collected using fluorescent microscope. The proliferation rates were estimated as the number of BrdU-positive cells per unit area and the mean proliferation rate for each group was calculated by averaging the rates of animals from animals in the same group (*n* = 4 mice/group; Encinas and Enikolopov, 2008). To analyze the stem cell and progenitors in the hippocampus, Nestin-labeled cells and GFAP-expression cells were calculated. Cell numbers were counted under the same conditions and photographed with identical microscope settings.

### Nissl's staining

The frozen sections were hydrated by rinsing into 100, 95, 90, 80, 70% alcohol and distilled water in order (5 min for each). Then the sections were stained in a 1% cresyl violet acetate solution for 5–10 min, and differentiated in 75% alcohol for seconds (pH = 4.10), then rinsed quickly in distilled water.

### RNA isolation, reverse transcription, and real-time PCR

Mice with stereotaxic injection and their control littermates were sacrificed 2 weeks after stereotaxic injection. Cell populations in the SVZ tagged with EGFP or tdTomato were separated by fluorescence activating cell sorting (FACS).Then, total RNA was extracted, and the miR-30c and semaphorin3A levels in the SVZ were assessed using real-time PCR. Spike-in miRNA, cel-miR-39 (Qiagen, Hilden, Germany), was added to the cell lysate before miRNA extraction to guarantee the reliability of endogenous references. Detailed process was performed as previously described (Peng et al., 2013). All primers sequences were designed using Primer 5.0 software (Premier, Canada) and synthesized in Invitrogen (Table 2). RNA samples were prepared from four independent samples (eight mice/group) and analyzed at least three times. For assessing miR-30c level, snord2 was selected to be as reference, and semaphorin3A results were normalized to the housekeeping gene *Gapdh*. All experiments were performed in triplicate.

**Table 2:** **Primers for quantification of miR-30c and semaphorin3A**.

**ID**	**Reverse Transcription primers (5′–3′)**	
miR-30c-5p	GTCGTATCCAGTGCAGGGTCCGAGGTATTCGCACTGGATACGACGCTGAG	
Snord2	GTCGTATCCAGTGCAGGGTCCGAGGTATTCGCACTGGATACGACAGTGATCAG	
cel-miR-39	GTCGTATCCAGTGCAGGGTCCGAGGTATTCGCACTGGATACGACCAAGCT	
ID	Forward Primer(5′–3′)	Reverse primer(5′–3′)
miR-30c-5p	GCCCGTCCTGTAAACATCCTACAC	CCAGTGCAGGGTCCGAGGTAT
cel-miR-39	CAGAGTAGCTCACCGGGTGTAAATC	CCAGTGCAGGGTCCGAGGTAT
Snord2	GGCAAATCATCTTTCGGGACTG	CCAGTGCAGGGTCCGAGGTAT
semaphorin3a	CCATTGTCAGCGCGTCTAGT	TAGCCGGTGGCTGACTCTAA
GAPDH	GAAGGTCGGTGTGAACGGAT	AATCTCCACTTTGCCACTGC

### Cell-cycles detection

Neuro2A cells were seeded in six wells (5 × 10^5^ cells/well), 24 h later, plasmids of miR-30c-OE, miR-30c-KD, and vehicle control were transfected into Neuro2A cells respectively (180 ng plasmids/well) with 1.8 μl lipofectamine 2000 (Invitrogen, CA, USA). 18 h post-transfection, the cells were harvested and incubated with propidium Iodide (PI, Sigma, 50 μg/ml) and RNAase A (Thermo, 10 μg/ml) for 30 min at room temperature (Hui et al., 2013). Then, these cells were analyzed by calculating 10,000 cells per sample *via* flow cytometry (FlowCytometer, Beckman Coulter, Brea, CA). Cell-cycle was analyzed using Wincycle 32 software (Beckman Coulter, Brea, CA).

### Statistics

Vector of vehicle control was served as the control for all the experiments in this study. All the quantitative data are presented as mean ± SD., the differences among groups were assessed by one-way ANOVA. *p* < 0.05 were considered to be statistically significant. Behavioral results with time and groups were analyzed by two-way ANOVA. All analyses were performed using SPSS (v.20.0, SPSS Inc., Chicago, IL).

## Results

### Changes of olfactory sensitivity by intervention of miR-30c in the SVZ

**Figure 1:**
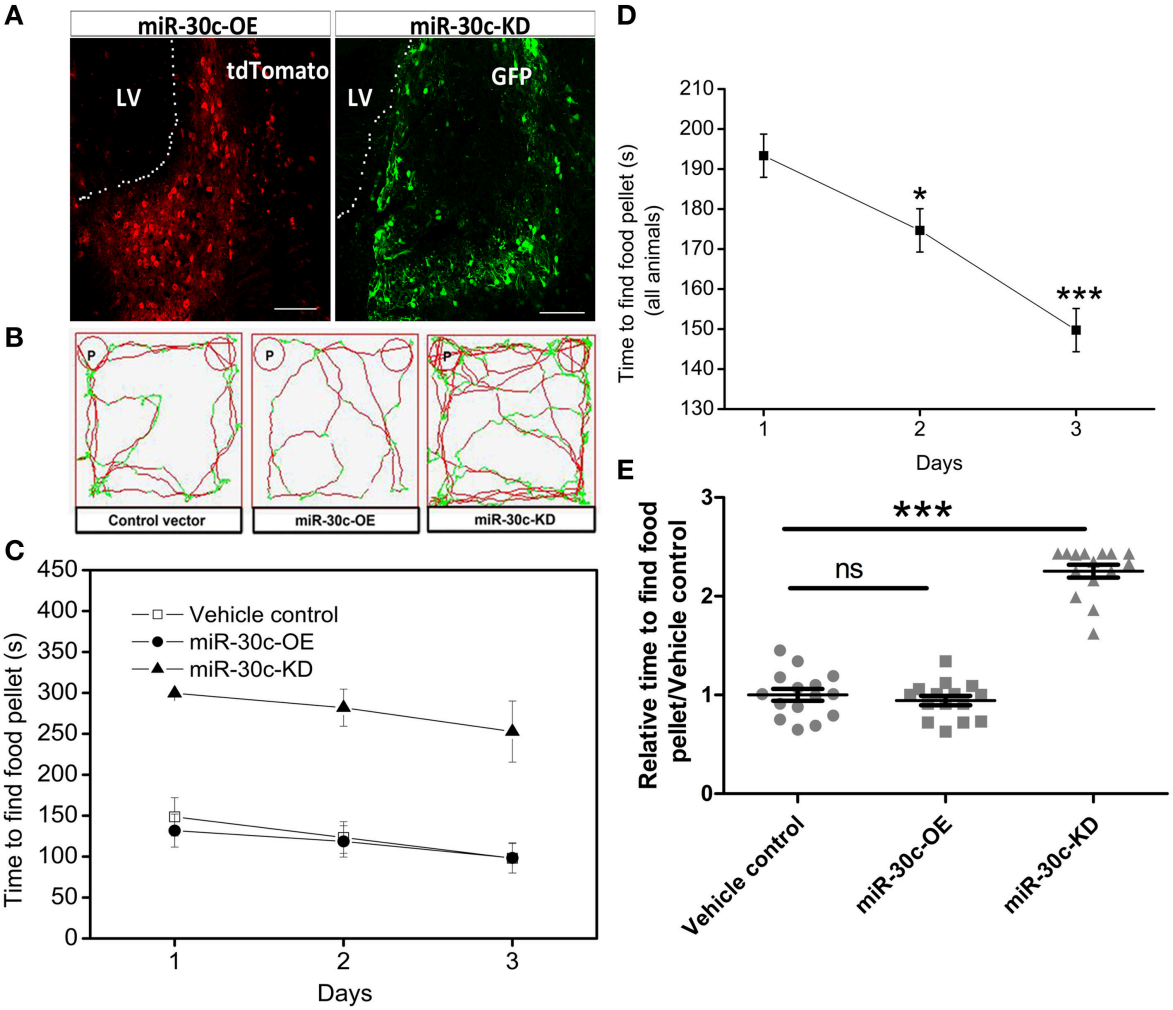
**Effect of miR-30c on olfactory sensitivity**. **(A)** Representative images of miR-30c-OE and miR-30c-KD expression in the SVZ 2 weeks after stereotaxic injection. Scale bar, 50 μm. **(B)** Trajectories of mice in finding food pellets detected by real-time infrared ray video. Red line, speed of mice > 10 cm/s and green line, speed of mice < 2 cm/s; p, food pellet location. **(C)** Time to find food pellets with days. **(D)** Time effect on olfactory sensitivity of all animals (results derived from two-way ANOVA analysis, time and groups as two main factors). ^*^p < 0.05, ^***^p < 0.001 determined by Bonferroni multiple comparison tests. **(E)** miR-30c effect on olfactory sensitivity in finding food pellets, ns, not significant; ^***^p < 0.001 determined by two-way ANOVA analysis followed by Bonferroni multiple comparison tests. miR-30c-OE, miR-30c overexpression; miR-30c-KD, miR-30c knockdown; LV, lateral ventricle**;** *n* = 15 mice, five mice/group.

To specifically regulate the level of miR-30c in the SVZ, we constructed the up-and down-regulated lentiviral vectors of miR-30c and tagged them with tdTomato (red) and GFP (green fluorescent protein), respectively. These fluorescent protein-tagged viral vectors were successfully expressed by integration into the genomes of the SVZ cells after stereotactic injection (Figure 1A).

To investigate the effects of miR-30c on olfaction, time of mice spent in finding the scented food was analyzed. In the food pellet buried experiment, the real-time trajectory and the speed of the mice (red: speed > 10 cm/s, green: speed < 2 cm/s) were recorded by infrared video. Mice in miR-30c-OE and vehicle control groups found and recognized the food location more quickly than miR-30c-KD mice (Figure 1B), and all mice found the pellets more rapidly with increase of training days [*F*_(2, 15)_ = 16.38, *p* = 6.36 × 10^−6^; Figures 1C,D], but miR-30c-KD mice needed more time to find buried pellets than vehicle control and miR-30c-OE mice (miR-30c-KD vs. vehicle control = 2.26 fold, *p* < 0.0001; miR-30c-KD vs. miR-30c-OE = 2.41 fold, *p* < 0.0001; Figure 1E). There was no significant difference in time consumed in finding the buried pellets for vehicle control and miR-30c-OE mice (miR-30c-OE vs. vehicle control = 0.94 fold, *p* = 1.00). Individuals in the same group did not differ in their ability to find the pellets [*F*_(5, 15)_ = 1.07, *p* = 0.4]. The interaction between groups and days was not significant [*F*_(4, 15)_ = 0.244, *p* = 0.911], indicating that mice from all groups improved similarly over time in their ability to find buried food pellets.

### OB morphological changes under the alteration of miR-30c in the SVZ

**Figure 2:**
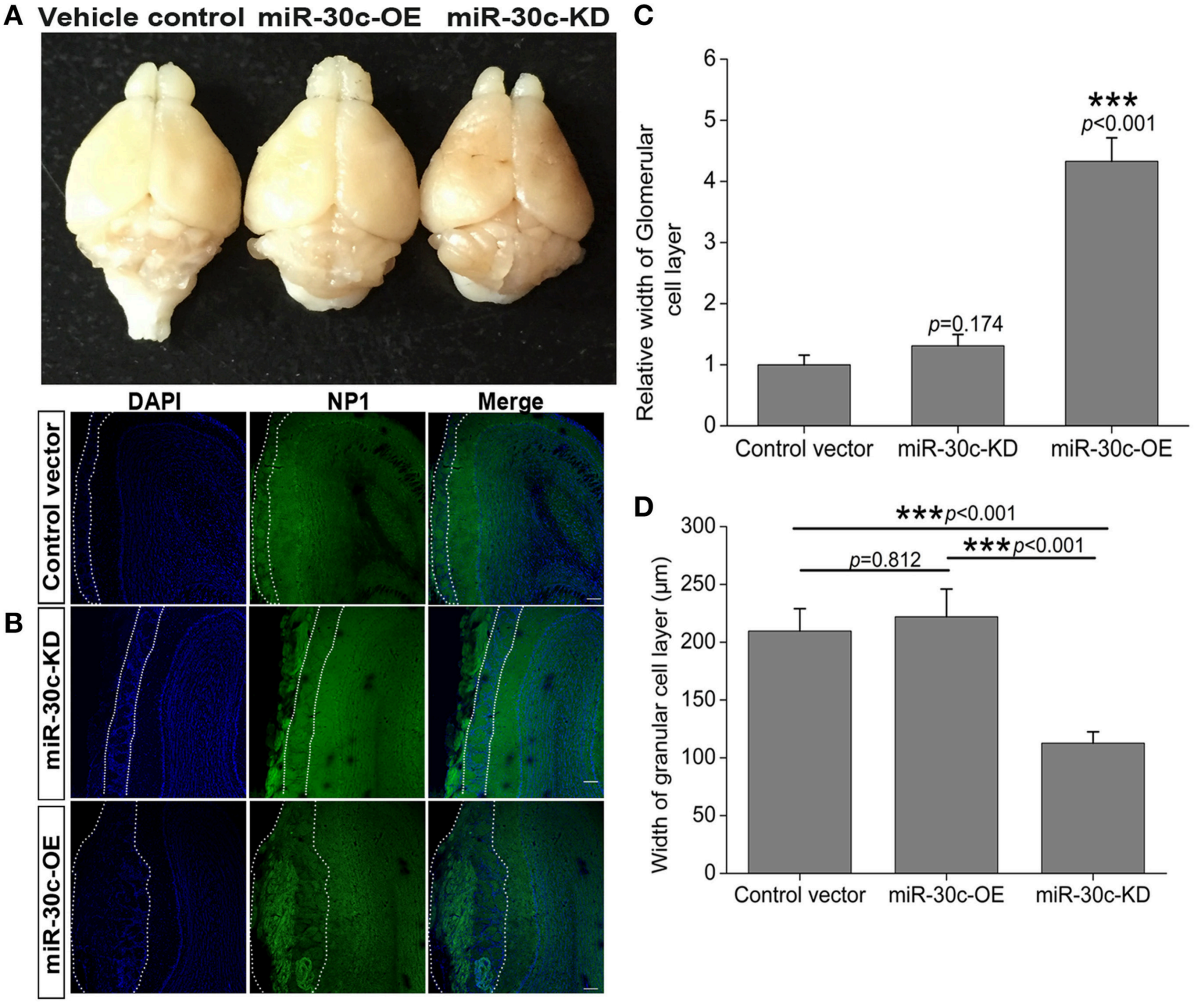
**Morphological alteration of the OB induced by changes of miR-30c level in the SVZ. (A)** The whole morphologies of OB 3 months after stereotaxic injection in the SVZ, *n* = 3 mice/group. **(B)** The glomerular cell layer and granular cell layer detection. Nuclei were stained by DAPI; Neuropilin-1(the receptors of semaphorin3A) expressed cells were stained with neuropilin (NP1). Regions between two dash lines were glomerular cell layer. Scale bar, 40 μm. **(C)** Statistic analysis width of glomerular cell layers. ^***^p < 0.001 determined by one-way ANOVA followed by Bonferroni multiple comparison tests. **(D)** Statistic analysis width of granular cell layers. ^***^p < 0.001 determined by one-way ANOVA followed by Bonferroni multiple comparison tests; *n* = 60 sections, four mice/group.

The above changes of olfactory sensitivity induced by miR-30c prompted us to detect the morphologies of the OB in all groups. After 3 months of stereotaxic injection, the OBs of miR-30c-KD mice were significantly smaller than vehicle control and miR-30c-OE mice. However, the OBs of miR-30c-OE and vehicle control mice were similar in volume (Figure 2A).

In addition, we also detected the different layers of the OBs in each group of mice. The width of glomerular layers in miR-30c-KD mice were significantly thinner than those of miR-30c-OE mice (miR-30c-OE vs. miR-30c-KD = 0.230 fold, *p* < 0.001; Figures 2B,C) and their granular cell layers were also significantly thinner than that of vehicle control (miR-30c-KD vs. vehicle control = 0.54 fold, *p* < 0.001). However, there was no difference in the width of glomerular layers between miR-30c-KD and vehicle control mice (miR-30c-KD vs. vehicle control = 0.762 fold, *p* = 0.174) and granular cell layers between miR-30c-OE and vehicle control were similar (miR-30c-OE vs. vehicle control = 1.06 fold, *p* = 0.812; Figures 2B,D). These results indicated that miR-30c alteration in the SVZ could give rise to the changes of the glomerular and granular cell layers in the OB.

### Changes of conditional fear memory by intervention of miR-30c in DG

**Figure 3:**
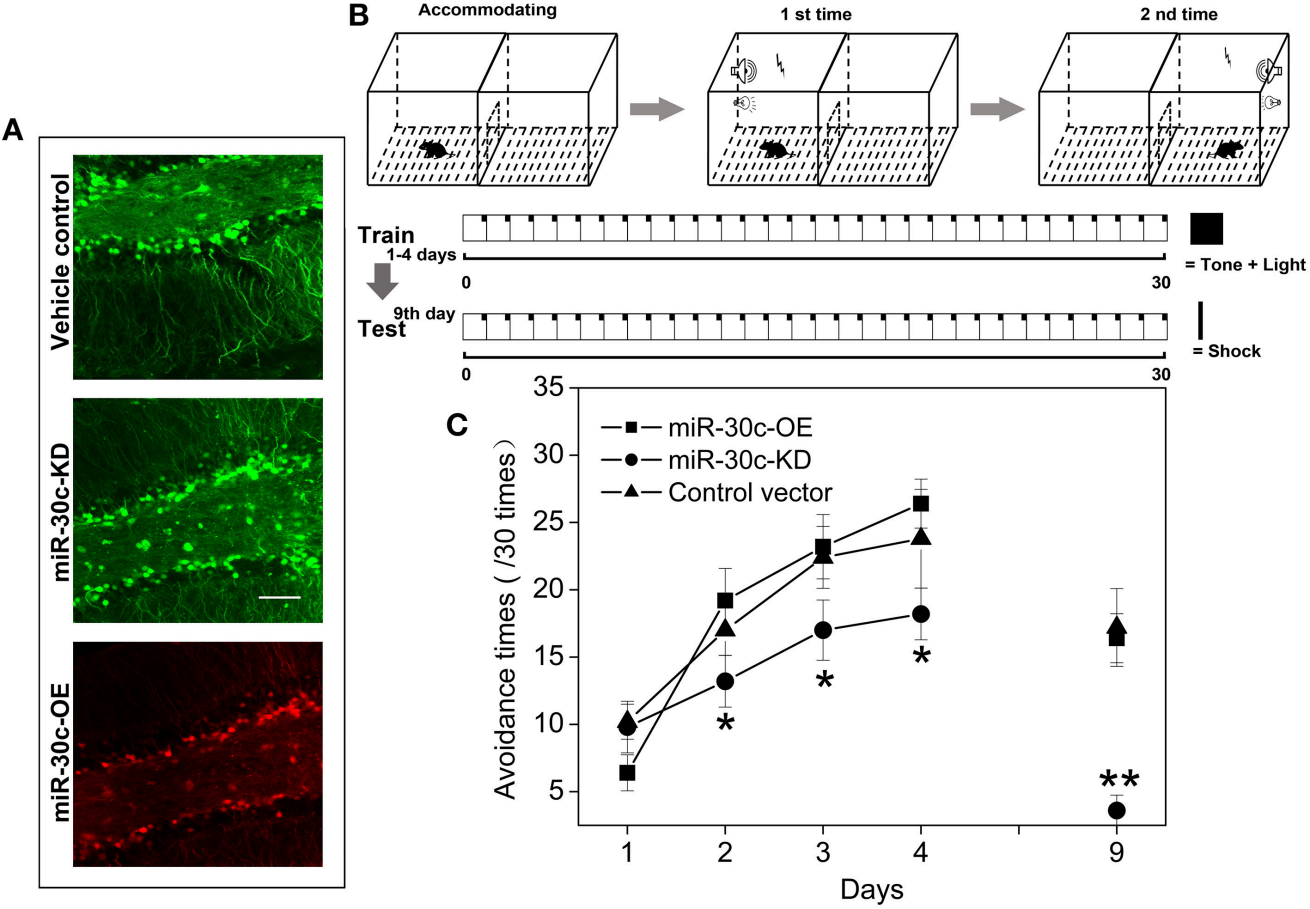
**Effect of miR-30c on associative memory. (A)** Representative images of miR-30c-OE and miR-30c-KD expression in DG 2 weeks after stereotaxic injection. Scale bar, 50 μm. **(B)** Schematics of conditional fear memory tests. Thirty cycles in one trial for one mouse each day. Firstly, mouse was accommodating in the test box for 5 min before test. Conditional stumuli (tone and light) gated by computer, were triggered exclusively in the chamber that the mouse stood on. Mouse escaped within 5 s, it would not be shocked, or it would be shocked until it escaped to the other chamber, the maximum shock time is 15 s. Both resulted in the termination of the cycle and the beginning of the next cycle. The total training time was 4 days with an interval of 24 h. Long-term memory test was performed on the 9th day. **(C)** Time effect on conditional fear memory. ^*^p < 0.05, ^**^p < 0.01 determined by two-way ANOVA analysis followed by Bonferroni multiple comparison tests; *n* = 15 mice, five mice/group.

Similarly in the SVZ, levels of miR-30c in the DG were interfered with by injection of the up-and down-regulated vectors. The tagged fluorescent protein expression indicated that miR-30c was under the regulation of miR-30c-OE and miR-30c-KD in the DG, respectively (Figure 3A).

To assess the effect of miR-30c on associative memory, the conditional fear memory test was performed, which is a good means for assessing the learning and memory performance associated with the hippocampus. As shown in Figure 3B, the number of shock escapes continuously increased for mice in all groups with training. It was notable that, from the second day of training, the shock escapes of miR-30c-KD mice were significantly fewer than that of the vehicle control, indicating the ability of contextual fear learning and memory of miR-30c-KD mice was weaker than that of the control. Whereas, there was no difference in the learning- and memory-ability of mice between the control and miR-30c-OE mice, reflected by the similar shock escapes during training days (Figure 3C).

### Changes of space memory by intervention of miR-30c in DG

**Figure 4:**
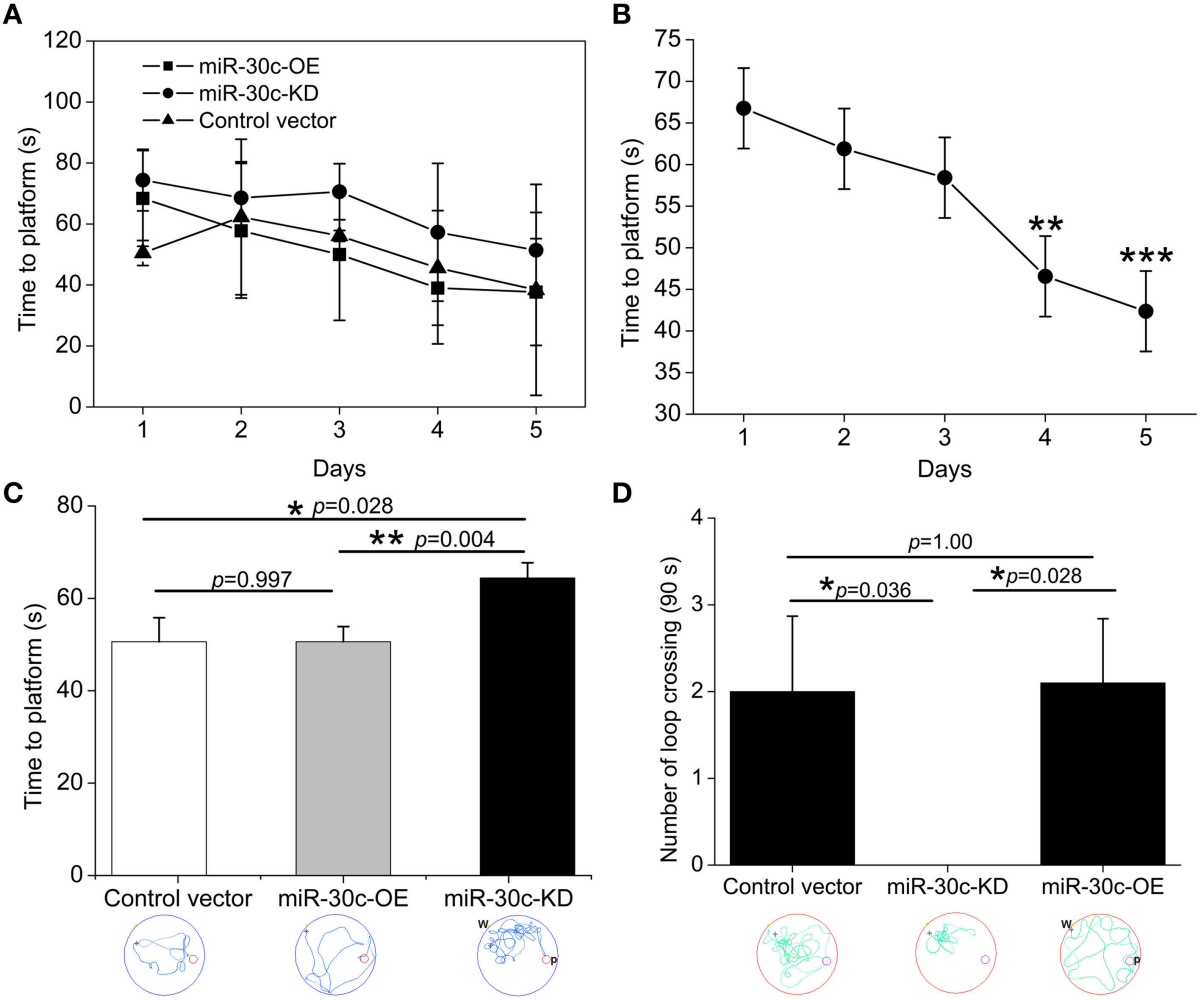
**Spatial learning and memory assessment by MWM test. (A)** Training-time effects on spatial learning and memory. **(B)** Statistic analysis the time effect on spatial performances of all animals. ^**^p < 0.01 and ^***^p < 0.001 determined by two-way ANOVA analysis, with the time and groups as two main effects. **(C)** Time to the platform location analysis within the five training days. ^*^p < 0.05 and *p* < 0.01 determined by one-way ANOVA followed by Bonferroni multiple comparison tests; blue lines in the circle, the trajectory of mice during training. **(D)** Times of crossing the platform location analysis on the 6th testing day. ^*^p < 0.05 determined by one-way ANOVA followed by Bonferroni multiple comparison tests; green lines in the circle, the trajectory of mice during test; W, place of entry; P, location of platform; *n* = 15 mice, five mice/group.

In the MWM test, escape latency (time to platform) and the number of crossings the immersed platform reflected the ability of spatial learning and memory. Results showed that the ability of spatial learning and memory improved with number of training days, represented as a descending tendency of time dependence in all groups especially on the 4th and 5th days (the 4th day vs. the 1st day = 46.6 s: 66.8 s, *p* < 0.01; the 5th day vs. the 1st day = 42.4 s: 66.8 s, *p* < 0.001; Figures 4A,B). We also found miR-30c-KD mice were weak in spatial learning and memory compared with the control and miR-30c-OE mice, reflected by an obvious increase in time to find the platform (*p* = 0.028 and *p* = 0.004, respectively). However, miR-30c-OE mice had no superiorities in spatial learning and memory than control mice (*p* = 0.997; Figure 4C). We measured the frequency of crossing the loop on the 6th day after training for 5 days. As shown in Figure 4D, the number of loop crossing for miR-30c-KD mice was obviously fewer than that of control and miR-30c-OE mice (*p* = 0.036 and *p* = 0.028), demonstrating the weakness of miR-30c mice in spatial learning and memory. But no difference was found between control and miR-30c-OE mice (*p* = 1.00).

### Neuronal lineage changes under the alteration of miR-30c in DG

**Figure 5:**
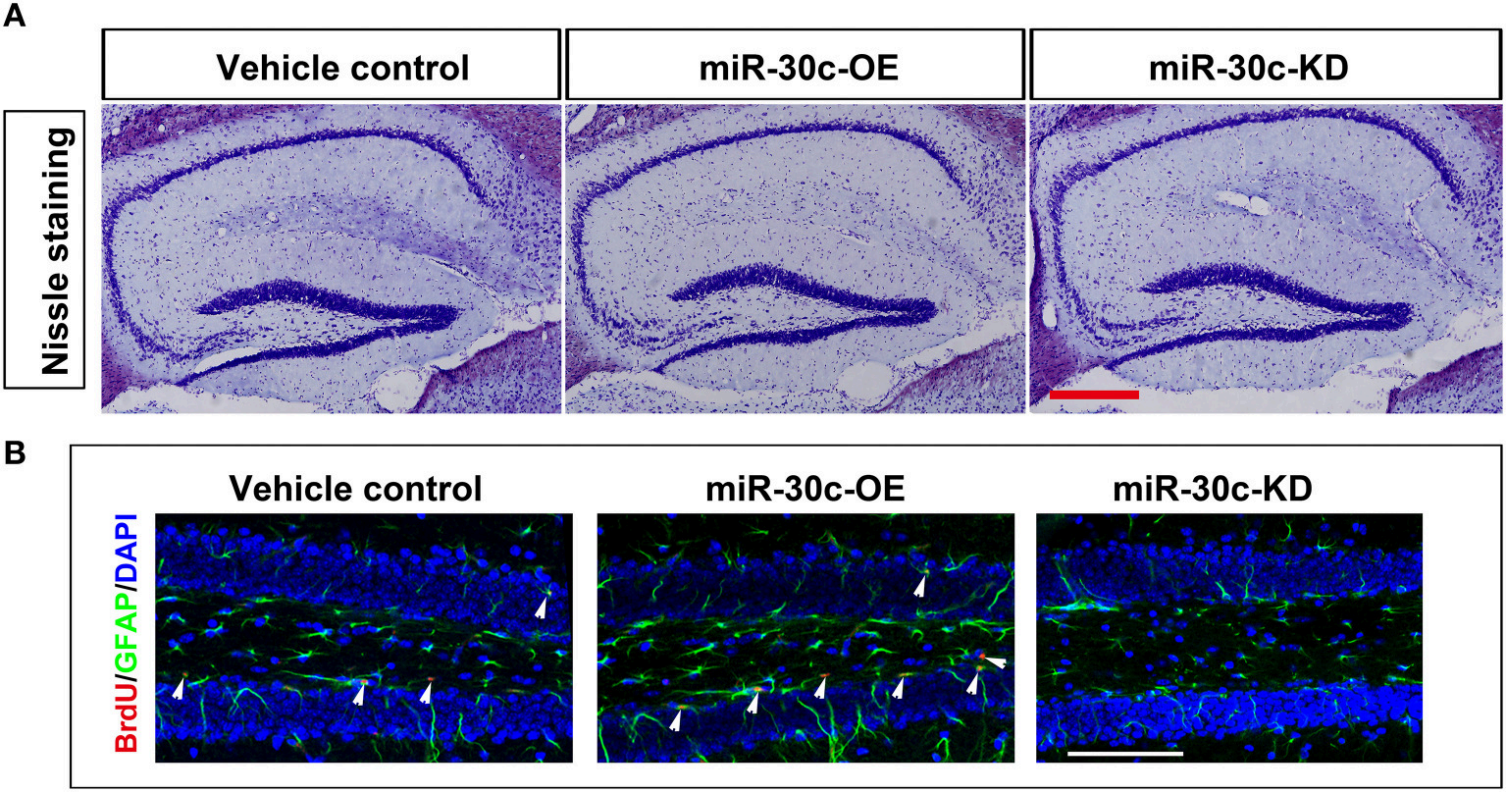
**Nissle staining and newborn cells' assessment in DG. (A)** DG whole morphology detection. Scale bar, 200 μm **(B)** Representative images of BrdU-labeled cell in DG by cumulative injection of BrdU for 7 days. Scale bar, 100 μm; *n* = 60 sections, four mice/group. Arrow heads, BrdU-labeled cells.

**Figure 6:**
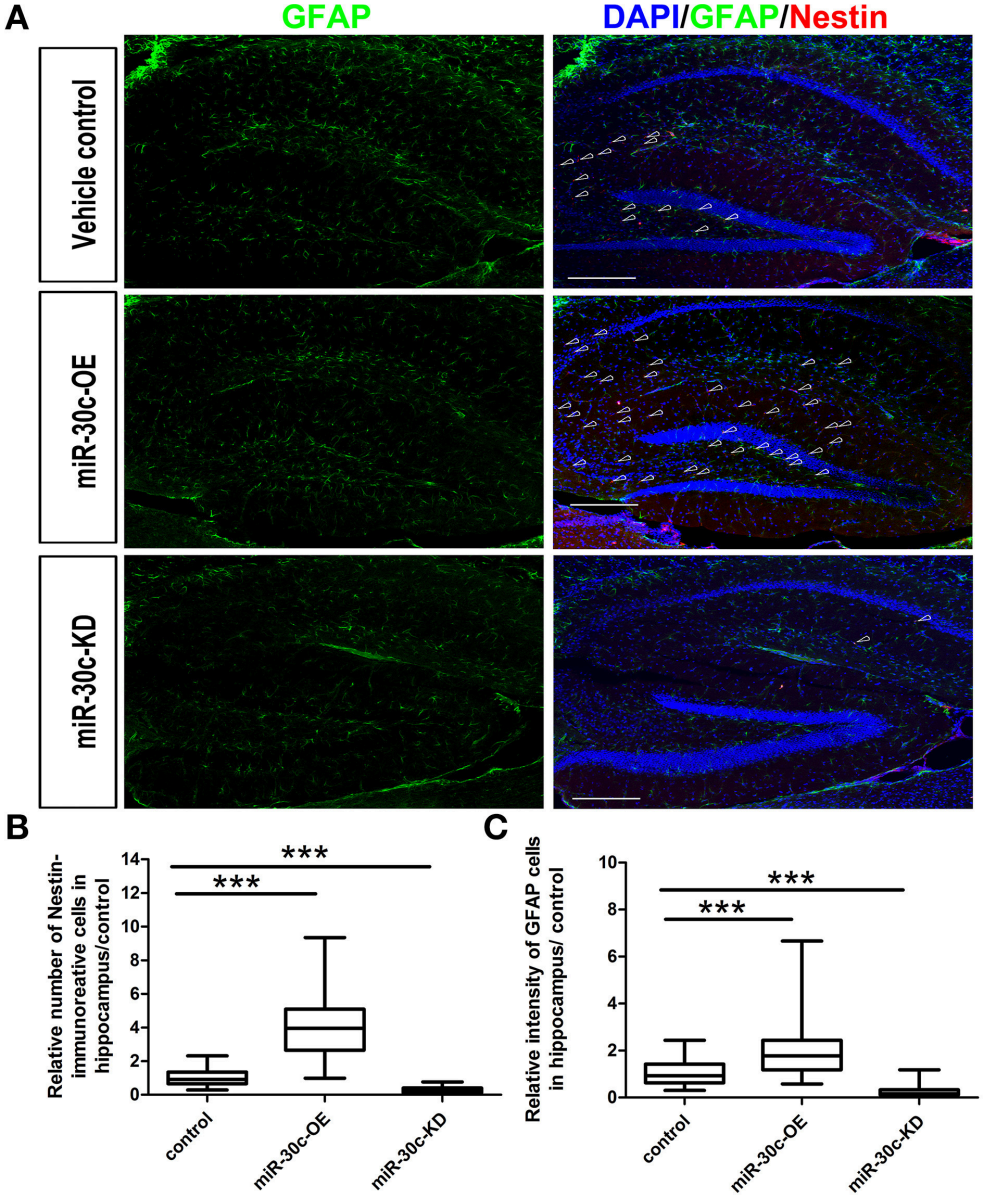
**Neuronal lineage alterations in the DG induce by miR-30c. (A)** Lineage alteration induced by miR-30c in DG. Nuclei were stained with DAPI; astrocyte, characterized with GFAP-immuoreactive cells; Stem cells were marked with Nestin-immoreactive cells. Scale bars, 100 μm. **(B,C)** Statistic analyses of the number of Nestin-immunoreactive (stem cells) and GFAP-immunoreactive cells (precursor of neuroblasts) in hippocampus. All data were normalized to the average value of the control, ^***^p < 0.001 determined by one-way ANOVA followed by Bonferroni multiple comparison tests; *n* = 60 sections, four mice/group.

We further investigated the hippocampus morphologies in each groups of mice. Nissle staining was employed for detection of the whole morphology of hippocampus and there was no morphological difference among the hippocampus of three groups (Figure 5A). To assess the effect of miR-30c on the newborn neurons, BrdU was used to label the newborn neurons 3 weeks after stereotaxic injection. Compared with that of the control group, there was an obvious increase in BrdU-labeled cells in the miR-30c-OE group and a decrease in the number of BrdU-labeled cells in miR-30c-KD group (Figure 5B). We also examined the effects of miR-30c on the neuronal lineage 2 weeks after BrdU injection. We found an estimated 4.08 ± 0.66-fold increase in the number of Nestin-immunoreactive stem cells in the DG of miR-30c-OE mice compared with the control and the miR-30c-KD groups (both *p* < 0.0001; Figures 6A,B). Furthermore, the miR-30c-OE group showed an increase of GFAP-positive astrocytes (miR-30-OE vs. vehicle control = 1.91 ± 0.40-fold, *p* < 0.0001), while the opposite was found in the miR-30c-KD group (miR-30c-KD vs. vehicle control = 0.28 ± 0.18-fold in nestin- immunoreactive stem cells; miR-30c-KD vs. vehicle control = 0.24 ± 0.23-fold in GFAP-positive astrocytes, both *p* < 0.0001; Figures 6A,C).

### Alteration of adult neurogenesis by miR-30c and semaphorin3A

**Figure 7:**
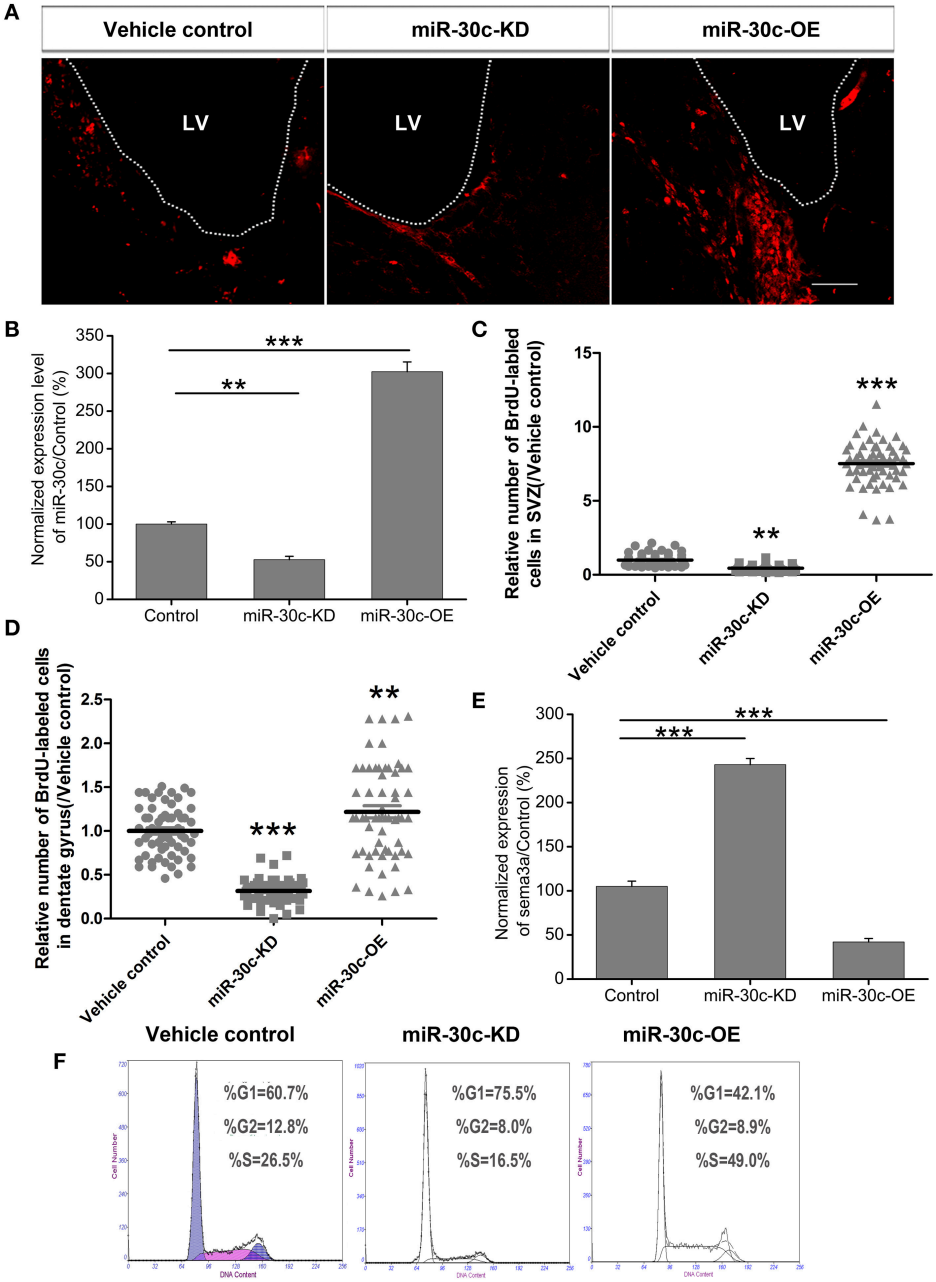
**Effect of miR-30c on adult neurogenesis by negative regulation of semaphorin3A. (A)** Representative images of newborn cells in the SVZ 4 h after intraperitoneal injection of BrdU. **(B)** Effects of miR-30c on adult neurogenesis. Expression of miR-30c was detected by qRT-PCR after cells sorted by fluorescence activating cell sorting (FACS), *n* = 8 mice/group. **(C)** Statistic analysis of BrdU-labeled newborn cells in the SVZ, 3 weeks after stereotaxic injection, *n* = 60 sections, four mice/group. **(D)** Statistic analysis of BrdU-labeled newborn cells in the DG, 3 weeks after stereotaxic injection, *n* = 60 sections, four mice/group. **(E)** Expression of semaphorin3A was detected by qRT-PCR. Data in **(B,E)** were analyzed by one-way ANOVA, ^**^p < 0.01 and ^***^p < 0.001 were determined by Bonferroni multiple comparison tests. Data in **(C,D)** were normalized to the average value of the control and analyzed by one-way ANOVA, ^**^p < 0.01 and ^***^p < 0.001 were determined by Dunnett T3 multiple comparison tests. **(F)** Cell-cycle assessment by flow cytometry, *n* = 3 wells/group.

To explore the basis for the structural and functional alteration of the OB and DG, we investigated the adult neurogenesis of these two adult neurogenesis regions by interfering with the levels of miR-30c and semaphorin3A expression. Statistics analysis of the BrdU-labeled cells showed that the adult newborn neurons increased to 8.69 ± 1.32-fold (*p* < 0.001), as the level of miR-30c in SVZ was elevated to 3.12 ± 0.031-fold (*p* < 0.001); In contrast, when the level of miR-30c was declined to 0.62 ± 0.013-fold (*p* < 0.05), the number of adult newborn neurons decreased to 2.03 ± 0.016-fold (*p* < 0.01; Figures 7A–C). Similarly, miR-30c elevation in the DG also increased the number of newborn neurons locally (1.22 ± 0.22-fold, *p* = 0.022). The opposite result was observed when miR-30c-KD was injected in the DG (0.31 ± 0.10-fold, *p* < 0.0001; Figures 5B, 7D).

We previously validated that semaphorin3A was negatively regulated by miR-30c in the OB (Sun et al., 2014). To detect whether semaphorin3A mediated in regulating adult neurogenesis under the regulation of miR-30c, we sorted the miR-30c-OE and miR-30c-KD expressing cells in the SVZ by flow cytometery *via* their respectively tagged fluorescence proteins. Quantitative results by qRT-PCR then showed that an elevated level of miR-30c in the SVZ gave rise to a decrease of semaphorin 3A (0.41 ± 0.012-fold, *p* < 0.001). Inversely, the level of semaphorin3A in the SVZ increased when miR-30c was lowered (2.48 ± 0.02-fold, *p* < 0.001; Figure 7E). The above results indicate that the promotive effect of miR-30c on adult neurogenesis was accomplished by negative regulation of semaphorin3A.

To further disclose the mechanism of miR-30c in modulating adult neurogenesis, we analyzed the cell-cycle of neuroblastoma, Neuro2A cells, under infection with miR-30c-OE and miR-30c-KD. Neuro2A is a mouse-neural-crest-derived cell line, endued with nearly all the attributes of neurons and it has been extensively used as neuron substitute in study of neuronal proliferation and differentiation (Schor et al., 2013; Fiszbein et al., 2016). In miR-30c-OE expressed cells, cell-cycling was stimulated, as shown by the increased proportion of cells in G2+M stages. In contrast, in miR-30c-KD expressed cells, cell-cycling was impeded by the increased proportion of cells in the G1 stage (Figure 7F). Taken together, miR-30c regulates adult neurogenesis by modulating the cell-cycle.

## Discussion

In this study, we investigated the effects of miR-30c on modulating the morphology and function of the OB and hippocampus through the regulation of adult neurogenesis. Strict regulation of this process is essential for maintaining a pool of newborn neurons for the ongoing functions of the OB and the hippocampus.

### Effects of miR-30c on morphology of the OB and hippocampus

The adult brain still preserves some neuronal regenerative regions. The SVZ and DG, two widely accepted neural stem-cell derived regions, continuously produce adult newborn neurons (Kriegstein and Alvarez-Buylla, 2009; Kelsch et al., 2010). Neural stem cells, astrocyte-like cells, are capable of generating neuroblasts and these neuroblasts migrate toward their target locations. Neuroblasts in the SVZ are capable of migrating along rostral migratory streams and finally integrating into the granule cell and periglomerular cell layers of the OB within 1–2 weeks (Song et al., 2005; Imayoshi et al., 2008; Lepousez et al., 2015). In addition, the integrated locations of these neuroblasts are dependent on the residence of neural stem cells in the SVZ. Evidence shows that neural stem cells from the medial wall of the lateral ventricle are preferentially differentiated into periglomerular cells (>85%), whereas those from the lateral wall are primarily differentiated to granule cells in the OB (>90%). In a third condition, neural stem cells located in the dorsal wall differentiated into periglomerular cells and granule cells, and the proportion of granule cells is 2/3 (Fiorelli et al., 2015). Here, stereotaxic injection sites are located at the medial wall of the lateral ventricle. We found the glomerular layer in miR-30c-OE OB was thicker than in the control, since the up-regulated miR-30c gave rise to an increase of adult born neurons in the SVZ (Figures 2B,C, 7A,C). While in the miR-30c-KD group, no difference was found in the periglomerular layer compared with control. These results are probably due to the fact that a limited small number of newborn neurons are needed for glomerular layer. Although, adult-born neurons were decreased significantly in miR-30c-KD mice, they were preferentially migrated and integrated into the periglomerular layer. In addition, the much thinner granule cell layer in the miR-30c-KD mice probably resulted from some leakage of lentiviruses into the dorsal wall during stereotaxic injection, which led to a proportion of the newborn neurons migrating into the granule cell layer (Figures 2B,D) Moreover, there is stringent regulation in newborn-neuron survival in the granule cell layer. The redundant newborn cells are not successfully survived during integration (Whitman and Greer, 2009). So, there was no marked difference in the granule cell layer between the miR-30c-OE and the control. When this effect lasted for 3 months, we found that the whole volume of the OB in the miR-30c-KD group was strikingly shrunk, whereas there was no significant difference in the miR-30c-OE and control groups (Figure 2A).

In the hippocampus, the neuroblasts from the DG migrated into the granular cell layer and were integrated into the local circuits within 4–10 days (Ming and Song, 2005; Figure 5B).The number of the adult newborn neurons in the DG is far fewer than that of the OB (Benarroch, 2013). So, alteration in levels of miR-30c in the DG does not give rise to whole volume changes in the hippocampus. However, an increase of adult born neurons and astrocytes was found in the miR-30-OE group. In contrast, newborn neurons and astrocytes in the DGs infected with miR-30c-KD were both reduced (Figure 6). Taken together, miR-30c has a direct effect on the whole morphology of the OB and the lineage constitution of the hippocampus through the regulation of adult neurogenesis.

### Effects of miR-30c on function of the OB and hippocampus

Adult neurogenesis is considered to be essential for morphological and functional maintenance of the OB circuit. The newborn neurons of the OB directly interact with the mitral cells, the other interneurons and the centrifugal fibers from the olfactory cortex, thus they play critical roles in the spatial and temporal output patterns of mitral cell activities (Macrides et al., 1981; Kiselycznyk et al., 2006; Wilson and Mainen, 2006; Gheusi and Lledo, 2007; Petzold et al., 2009; Strowbridge, 2009). The functional disclosing of adult neurogenesis is one of foci in neuroscience (Oboti et al., 2011; Giachino and Taylor, 2014; Mohn and Koob, 2015). Studying the effects of certain genes on olfactory behavior by genes knockout often results in conflicting data, because the effects of genes on the whole body or compensatory effects elicited during development (Enwere et al., 2004; Kim et al., 2007; Bath et al., 2008).

Here, we specifically intervened in the level of miR-30c in the SVZ and the DG by stereotaxic operation, achieving location-specific alterations of miR-30c. We found the amount of newborn neurons correlated closely to the level of miR-30c, and inhibition of adult neurogenesis resulted in the reduced olfactory sensitivity. Whereas, Imayoshi et al. reported that the ablation of adult-born neurons did not affect olfaction using conditional knockdown transgenic mice. This discrepancy is probably due to the differences in affected neuronal lineage and detection odors. The nestin-knockout transgenic method mostly exerted on the glial fibrillary acidic protein-expressed cells (85.1 ± 0.4%), whereas, doublecortin-expressed cells and a half of S100β-positive cells in the SVZ are not affected (Imayoshi et al., 2008). In our study, cells that interfered with were not limited to nestin-immunoreactive stem cells, other stem cells or neuroblasts were also under affection, due to the extensive infection of lentiviruses. Since these stem cells mature and integrated into different locations in the OB, which probably led to differences in functions. In addition, the odors used for olfactory detection are different. Simple odors were used in nestin-transgenic mice, while a compound odor-grape cookie was used here. In this study, we also found the redundant newborn neurons could not further improve olfactory sensitivity, though significant morphological changes in the glomerular layer appeared (Figures 1C,E, 2B,C).This is probably due to the saturation of the olfactory circuits or that the olfactory sensitivity is more dependent on the type of newborn neurons rather than their number.

Adult newborn neurons in the hippocampus are derived from the subgranular zone (Dhaliwal and Lagace, 2011). It estimated that about 1400 newborn neurons are added daily to the bilateral hippocampi of the human adults. The number accounts for ~1.8% of the total renewable neuronal populations (Spalding et al., 2005). Though the hippocampus has a limited number of adult newborn neurons, each adult born neuron is estimated to make contact with ~12 CA3 pyramidal neurons. These CA3 pyramidal cells further communicate with 40–60 neighboring pyramidal neurons and 20–30 adjacent inhibitory cells. Thus, the neuronal response within the hippocampus is amplified (LeBeau et al., 2005). Besides, the adult born neurons have lower induction threshold for long-term potentiation, which increases the intrinsic excitability (Schmidt-Hieber et al., 2004). Using the retroviral labeling, studies validated that the adult newborn neurons have direct synaptic connections with the granule neurons with primary innervations from the lateral entorhinal cortex which is the core of the cued and contextual information processing region (Marín-Burgin et al., 2012; Vivar et al., 2012; Vivar and van Praag, 2013). The newborn neurons play important roles in the forming and processing of memory by synaptic connection with CA3 pyramidal cells (Toni et al., 2008).

Through specific interference in the level of miR-30c in the DG, we found the level of miR-30c positively correlated with the number of newborn neurons in the DG (Figure 7D). Fear-memory tests showed ability of contextual fear memory was declined in the miR-30c-KD group as adult neurogensis was reduced. However, no significant differences were found in the miR-30c-OE group in fear-memory compared with the control group (Figure 3C), indicating that the synaptic connections between new born neurons and CA3 pyramidal cells are saturated, the superfluous newborn neurons cannot form synaptic connection, where the positive feedback cannot further enhanced (Toni et al., 2008). On the 9th testing day, a general decline in fear-memory occurred in all groups. But the miR-30c-KD group had a quicker decline compared with that of control group and miR-30c-OE groups (Figure 3B), suggesting that newborn neurons participate in the long-term memory process (Wang et al., 2014). Moreover, similar results appeared in the spatial memory tests. Taken together, the newborn neurons are not only involved in associative memory, but also play critical roles in spatial learning and memory (Snyder et al., 2005), which can be interpreted by the fact that after the MWM training, distinct expressional profile was found in the newborn neurons compared with the normal newborn neurons without training. Besides, the newborn neurons have lower long-term potentiation, indicating that these newborn neurons are activated during training (Ramirez-Amaya et al., 2006; Kee et al., 2007).

In conclusion, our study shows that changes expression of miR-30c in the SVZ and DG induces changes of newborn neurons in respective regions, which results in the morphological changes of the OB and lineage constitutional alteration in the hippocampus. Deficiencies of adult-born neurons in the SVZ and DG led to abnormalities in morphology and function, suggesting a certain number of newborn neurons is necessary for morphological and functional maintenance of the brain. However, regional and lineage specific methods should be developed to further disclose the functions of adult neurogensis and the significance of redundant newborn neurons in the brain.

## Author contributions

TS and SL conceived and project and designed the experiments; TS, TL, WL, JY, and SL performed and analyzed the experiments on behavioral tests; TS and TL performed and analyzed the experiments on immunohistochemistry, cell-cycles detection and stereotaxic operations. TS wrote and revised the manuscript; HD gave good suggestion for research direction and revision of the manuscript. All the authors made a critical revision for the manuscript and all approved the final version of the manuscript.

## Funding

This work was supported by the National Natural Science Foundation of China (81371404 and 81571243).

### Conflict of interest statement

The authors declare that the research was conducted in the absence of any commercial or financial relationships that could be construed as a potential conflict of interest.
